# A New Playbook: State‐Driven Solutions for Resilient Health Data

**DOI:** 10.1111/1468-0009.70103

**Published:** 2026-06-22

**Authors:** NINEZ A. PONCE, RITI SHIMKHADA, TARA BECKER, SUSAN BABEY, A.J. SCHEITLER

**Affiliations:** ^1^ UCLA Center for Health Policy Research; ^2^ UCLA Fielding School of Public Health

**Keywords:** data collection, population characteristics, racial groups, health equity, policy, federal government, state government, United States

## Abstract

**Policy Points**

- Disruptions to federal health data infrastructure threaten states’ ability to identify health disparities, target interventions, and evaluate programs, making state‐level investment in data infrastructure and systems especially urgent.
- States can build resilient, equity‐centered data systems by enacting data disaggregation legislation, investing in data systems with bridging methodologies for data continuity, and establishing governance frameworks that protect privacy while facilitating data access.

**Context:**

Health equity depends on data equity: the representation of communities in the data used to identify disparities, target interventions, and hold systems accountable. Recent shifts in federal health data infrastructure, including changes to datasets and reductions in agency capacity, have introduced uncertainty for state officials who bear primary responsibility for population health outcomes. Even apart from these shifts, robust state capacity matters in its own right, making investment in resilient data infrastructure a timely priority.

**Methods:**

We examine how changes in federal health data infrastructure affect states’ capacity to advance data equity and how policymakers can respond. We review across state data disaggregation legislation, draw on the California Health Interview Survey (CHIS) as a model case, and synthesize evidence on data interoperability, governance, and dissemination.

**Findings:**

Some states are already moving beyond federal minimums on race and ethnicity data collection. For example, Connecticut, Oregon, and Massachusetts have enacted expansive laws collecting detailed subgroups; Illinois, New York, and New Jersey have added Middle Eastern and North African and other categories across all state agencies. Colorado and Oregon extend collection to sexual orientation, gender identity, and disability status. We offer examples from California's recent legislation and show how CHIS operationalizes such mandates. We also identify persistent constraints, including incomplete demographic fields in administrative data, workforce and interoperability gaps, and the need for governance frameworks and data firewalls that safeguard privacy.

**Conclusions:**

State‐led data infrastructure will help build effective public health practice. The aim is not 50 incompatible systems but an ecosystem of comprehensive and inclusive systems that are more granular, responsive, and community‐accountable. For such an ecosystem to function, the systems must still communicate, which depends on shared standards and definitions that keep data comparable across states and over time. Realizing this vision requires diversified funding, cross‐agency coordination, strong governance, and active roles for researchers, philanthropy, and communities, so that progress in measuring disparities endures across changing policy environments.

Recent disruptions to federal health data infrastructure, including the alteration of nearly half of all tracked federal data sets in early 2025, have created an urgent “data vacuum” for state health officials. While this federal volatility makes state‐level investment a necessity, the need for robust state capacity also exists independently of these shifts. States hold primary responsibility for program outcomes and are uniquely positioned to move beyond federal “floors” to create equity‐centered systems. By leveraging their proximity to communities and their specific jurisdictional authority, states can implement data standards that go beyond federal standards to make marginalized populations visible and ensure that data are contextually relevant to local policy needs.

Effective policy responses to disease burden, mortality, and life expectancy challenges require an infrastructure for data that makes population health outcomes visible across demographic groups and accessible to policymakers, researchers, and community members to spur action.^1^ Such infrastructure is the foundation of data equity, or the fair representation in and access to data for all communities.^2^, ^3^, ^4^, ^5^, ^6^ The infrastructure required for data equity goes beyond data collection and data storage as it includes the systems for dissemination, the standards and definitions that enable comparability across sources, the governance structures that determine what is measured and who has access, and the human and digital capital required to maintain and interpret these systems.^6^, ^7^, ^8^ Without this infrastructure, policymakers cannot identify which populations face the greatest burdens, target interventions efficiently and effectively, measure progress, or hold systems accountable.

Data equity scholars argue we will not achieve health equity without data equity largely because the populations most affected by health inequities are often those most likely to be invisible in health data.^3^, ^4^, ^9^, ^10^, ^11^, ^12^ Invisibility is the result of insufficient sample sizes for small populations, multilingual communities being excluded by English‐only surveys, and the use of broad categorizations that obscure the experiences of people at intersections of multiple marginalized identities.^5^, ^13^, ^14^, ^15^ This paper examines how recent disruptions to federal health data infrastructure affect states’ capacity to advance data equity and identifies what policymakers can do to build systems that can withstand such disruptions. We focus on three critical infrastructure needs: the technical architecture for data systems, standards and shared population definitions, and mechanisms for data democratization. We offer examples of legislative actions from states such as California, Colorado, and Oregon that have advanced the goal of building state data infrastructure to collect data on race/ethnicity, sexual orientation and gender identity (SOGI), and disability. We draw on the California Health Interview Survey (CHIS) as a model for states that want to continue to support and build data infrastructure in the absence of federal support.

## The Federal Data Infrastructure Landscape

**Figure 1:**
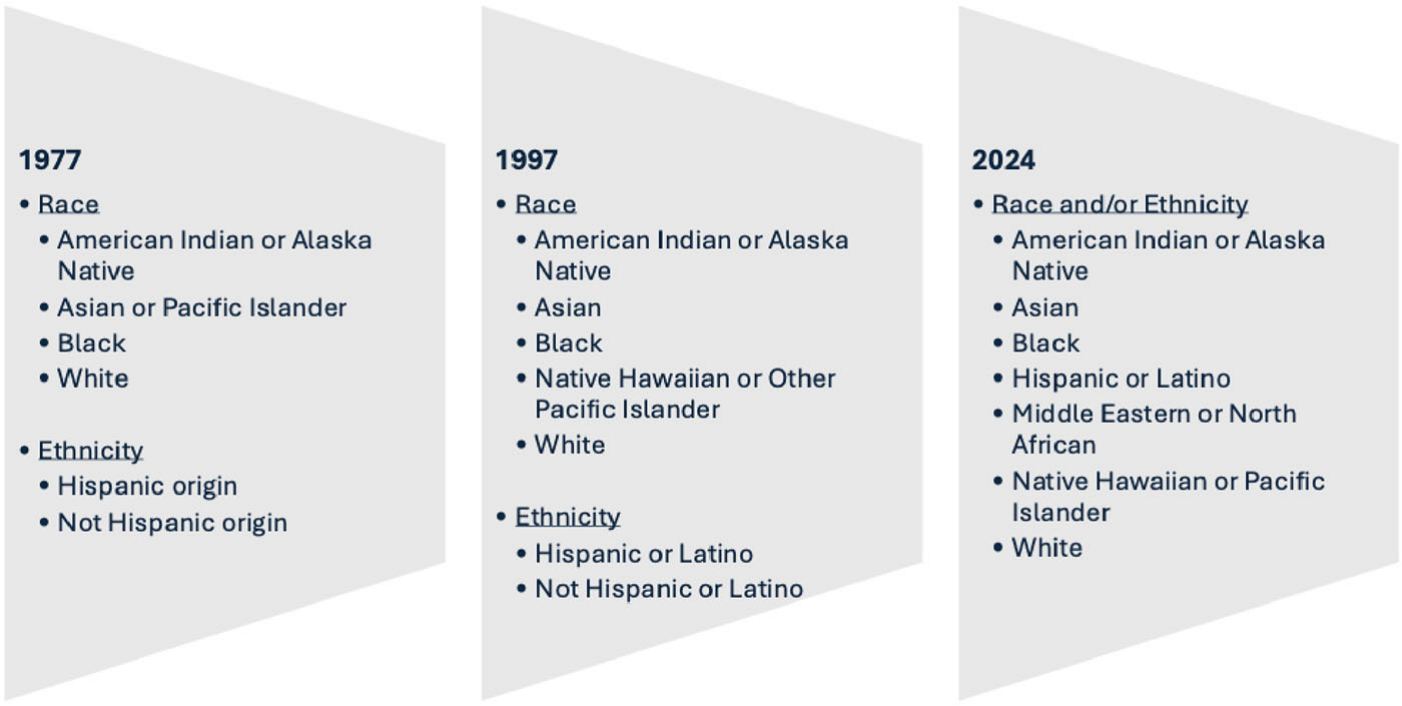
US Office of Management and Budget's Statistical Policy Directive No. 15 (SPD 15) Race and Ethnicity Standards Over Time [Colour figure can be viewed at wileyonlinelibrary.com] *Note*: For the 1997 and 2024 standards, an individual can select more than one race.

The US government has historically played three essential roles in health data infrastructure: standard setting, funding and coordination, and direct data collection.^1^ The US Office of Management and Budget's (OMB) Statistical Policy Directive 15 (SPD 15), first issued in 1977,^16^ revised in 1997,^17^ and revised again in 2024,^18^ established minimum categories for federal data collection on race and ethnicity (Figure 1).^2^ These standards, born from Civil Rights Act mandates to monitor discrimination, provided the definitional foundation that enabled comparability across federal surveys, state data systems, and administrative records.^6^ When a state health department calculates population statistics, for example, diabetes prevalence by race, it often relies on definitions that align with US Census Bureau population estimates.^19^ Critically, SPD 15 represents a floor, not a ceiling: states may collect race and ethnicity data that are more detailed and comprehensive than federal requirements.

Federal coordination in public health data has historically extended beyond definitions to methodological guidance. The Behavioral Risk Factor Surveillance System exemplifies this coordinating function: states implement standardized surveys with methodological support from the Centers for Disease Control and Prevention, enabling comparative analyses across states.^20^ Other federal data sources including the National Health Interview Survey, American Community Survey (ACS), and vital statistics systems have provided sampling frames, population benchmarks, and annual data that allow for tracking disease burden over time.^21^

Federal data sources, particularly the ACS and US Census Bureau population estimates, serve as the essential denominators for health statistics. When public health researchers calculate disease rates, mortality statistics, or health disparities, they divide health events (e.g., numerators from survey data) by population counts (denominators from federal data). Without accurate, demographically detailed population denominators, it is impossible to determine whether a community experiences higher rates of disease, whether health outcomes are improving over time, or whether interventions are effective.

The US Census Bureau's Population Estimates Program uses data on births, deaths, and migration in combination with decennial census data to produce annual population estimates at national, state, county, and subcounty levels by age, sex, race, and ethnicity. These estimates serve as denominators for calculating mortality rates by the National Center for Health Statistics, cancer incidence and survival rates by the National Cancer Institute's Surveillance, Epidemiology, and End Results program, and nearly all disease surveillance systems. The ACS provides additional demographic details on income, education, employment, health insurance coverage, language, and disability, all of which support understanding how social determinants of health vary across population groups and targeting interventions. Age‐adjusted mortality rates enable comparing health outcomes across populations with different age structures. These require age‐specific population denominators. Life expectancy calculations depend on age‐specific mortality counts combined with population estimates. Health disparity metrics require race‐ and ethnicity‐specific denominators and outcomes to determine whether certain communities experience disproportionate disease burden. Survey weighting and calibration procedures use federal population estimates to ensure that health surveys, including Behavioral Risk Factor Surveillance System and state surveys like CHIS, produce estimates representative of their sampled populations.

When federal demographic data are altered, delayed, or discontinued, these foundational statistics become unreliable or impossible to calculate. A 2025 study tracking 232 data sets from the Department of Health and Human Services, Centers for Disease Control and Prevention, and Department of Veterans Affairs between January 20 and March 25, 2025, found that 49% were altered.^22^ Of these altered data sets, 93% had terminology changes from “gender” to “sex,” and 78% had data classification or categorization altered, although only about 13% of these alterations were recorded in public documentation.^22^ Other disruptions include the indefinite halt of the Pregnancy Risk Assessment Monitoring System and the removal of racial and ethnic data from the 2024 National Survey on Drug Use and Health Annual Report.^23^ Beyond data removal, the disruption extends to infrastructure itself. Federal funding cuts have reduced federal agency capacity for technical assistance to states, maintenance of data systems, and methodological innovation. When federal agencies lose capacity, states lose data as well as the coordination architecture that enabled comparability across states and over time. This means dashboards that depend on federal data inputs, such as America's Health Rankings, lose functionality as data disappears. Preservation efforts are essential for maintaining access to historical data, but archiving cannot substitute for ongoing data generation. Communities and policymakers need up‐to‐date data to understand emerging health threats, document changing disparities, and evaluate policy interventions.

## Building State Data Infrastructure: Lessons From Multiple States

The federal data vacuum creates both urgency and opportunity for state leadership. States possess authority to collect and share disaggregated health data, structural advantages including proximity to communities, direct stakes in program outcomes, and increasingly the technical capacity to build sophisticated data systems. A scan of state laws in 2023 showed that 13 states had passed laws requiring the disaggregation of race and ethnicity data beyond the requirements of the 1997 SPD 15: California, Connecticut, Hawaii, Illinois, Massachusetts, Minnesota, Nevada, New Mexico, New York, Oklahoma, Oregon, Rhode Island, and Washington.^24^ States are building data infrastructure through several mechanisms: mandating that state agencies collect detailed demographic information on forms and surveys, requiring hospitals and health care providers to gather patient‐level race and ethnicity data at discharge, establishing standardized data collection frameworks, and creating oversight bodies such as health equity offices or racial equity advisory panels to coordinate collection efforts. States with existing disaggregation laws tend to build on them incrementally, expanding requirements to additional populations or agencies over time, suggesting that initial legislation creates institutional capacity and momentum for more comprehensive data systems.^24^

States have taken various approaches to expanding data collection beyond federal minimums.^25^ For example, Connecticut, Oregon, and Massachusetts have enacted expansive laws, each requiring collection of data on more than 35 race and ethnicity subgroups. Connecticut's 2021 (Public Act [PA] 21‐35) law requires state agencies collecting data for health care or public health purposes to ask patients to self‐identify from more than 60 race and ethnicity categories.^26^ Oregon's 2013 Race, Ethnicity, Language, and Disability (REALD) law requires the Oregon Health Authority (OHA) and Department of Human Services to collect detailed demographic data from health program applicants and health care providers, originally including 39 race and ethnicity subgroups that expanded to 72 categories in a 2024 update aligned with the revised federal SPD 15 standards.^27^ To support implementation, OHA maintains a dedicated full‐time staff team funded by the state's general fund, has integrated data collection into the Medicaid eligibility system, and provides training to help health care providers modify their electronic health records.^28^ In its 2024 budget, Massachusetts enacted a data equity mandate that requires all state agencies to collect disaggregated data on major Asian, Pacific Islander, Black, Latino, and White subgroups, notably applying to all state agencies rather than only health‐related agencies and departments.^29^ Other notable examples include Illinois's 2023 law (HB 3768), which made it the first state to require a Middle Eastern or North African (MENA) category, applying to all state agencies. New York adopted laws requiring collection of both Asian American and Native Hawaiian and Pacific Islander (NHPI) subgroups (2023) and MENA subgroups (effective July 2026), both applying to all state agencies.^30^ New Jersey's 2024 law requires state agencies to disaggregate data for Asian American, NHPI, MENA, and South Asian and Indian Diaspora communities (e.g., Guyanese).^31^

State experience suggests that providing more options for racial and ethnic self‐identification enables greater data disaggregation, which becomes especially important as populations grow more diverse. Such changes require coordination and technical assistance. Since March 2024, the California Department of Public Health Data Disaggregation workgroup has brought together departments, community‐based organizations, researchers, and data analysts in monthly meetings to help agencies integrate various standards and address implementation challenges. For example, Assembly Bill (AB) 1726 (2016) required disaggregated data collection for Asian American and Pacific Islander communities, but agencies have struggled to comply with it for various reasons, many of which stem from lack of technical support on how to implement changes.^32^ California's 2024 Latino and Indigenous Disparities Reduction Act (Senate Bill [SB] 435) is set to extend similar disaggregation to Latino communities, including Mesoamerican Indigenous identities and languages, such as Maya, Mixteco, Zapoteco, and Triqui.^33^ California's 2025 MENA Inclusion Act (AB 91) requires state and local agencies to collect and report disaggregated demographic data for MENA communities beginning January 1, 2027. Notably, the AB 91 definition of MENA departs significantly from federal standards by including several ethnic subgroups classified as White, Asian, or Black under federal guidelines. For example, under AB 91, the Armenian population is included as a “Transnational Middle Eastern and North African” group, alongside Amazigh, Assyrian, Chaldean, Circassian, and Kurdish populations.^34^ This differs significantly from federal OMB standards, which currently classify Armenian people as part of the White classification rather than as a MENA subgroup. California's decision to include the Armenian population in a MENA subgroup reflects both the state's large Armenian population and advocacy from community organizations arguing for recognition of cultural and geographic ties to the region.

Beyond expanding demographic categories, effective state data infrastructure requires interoperability, or the ability of two or more systems to exchange information and use the information that has been exchanged.^1^, ^35^ The COVID‐19 pandemic revealed major gaps in public health agencies’ data and reporting infrastructure, including outdated information systems that did not work well with other systems, data organized in nonstandard ways, and data‐sharing policies that were not flexible.^36^ A 2025 scoping review of over 20 studies on public health data modernization identified persistent challenges including data quality issues, lack of interoperability, and limited resources, while noting that best practices involve transitioning to cloud‐based systems, consolidating fragmented data into unified platforms, applying governance frameworks, and implementing analytics tools to support decision‐making.^37^

State data infrastructure also requires investment in specialized workforce capacity. The importance of dedicated technical staff was demonstrated during the COVID‐19 pandemic, when jurisdictions with robust data systems and in‐house expertise were better positioned to respond quickly to changing data needs. New York City's death reporting system performed effectively during the pandemic surge precisely because the city had invested substantially in technology and maintained its own information technology and scientific personnel who could rapidly deploy needed changes.^38^ In contrast, jurisdictions lacking such investments struggled to report timely data.

## The CHIS: A Model for State Data Infrastructure

The CHIS demonstrates that states can support building data infrastructure. Operating for over two decades since 2001, CHIS was designed to illuminate health disparities through community‐engaged, equity‐centered data collection. With approximately 20,000 household respondents annually, CHIS provides representative population estimates at the state level, for large and medium counties, and for racial and ethnic subgroups. Comprehensive state health surveys like CHIS require sustained funding investments. Diversified funding sources can enable them to weather changes in economic and political environments. CHIS has been funded by local, state, and federal agencies; nonprofit organizations; and private foundations. Major funders include state entities, such as the California Department of Health Care Services, California Department of Public Health, Covered California, and First 5 California. The state of California's ongoing commitment to CHIS across several decades, including during periods of economic downturns, underscores the state's recognition that population health surveillance requires sustained public investment beyond one‐time appropriations. Private foundations are also major contributors, including the California Endowment, California Wellness Foundation, and California HealthCare Foundation. This diversified funding model provides both stability and flexibility to develop modules and sampling approaches to address emerging health priorities and populations. For example, during the COVID‐19 pandemic, philanthropic support enabled rapid expansion of data collection.

CHIS's sampling and data collection strategies explicitly address the challenge of small population representation. Oversampling of American Indian and Alaska Native (AIAN) populations in 2001 and 2011‐2012, using Indian Health Service enrollment data supplemented by tribal community input, provided more robust data on California's AIAN population than federal surveys typically achieve.^39^ CHIS also allows respondents to identify as more than one race, which allows for detailed disaggregation of the multiracial population. This is crucial because half or more of AIAN and NHPI individuals identify with multiple races. By capturing multiracial identification and allowing research to examine these populations both separately and in combination with single‐race groups, CHIS enables a more nuanced understanding of multiracial health experiences to emerge while preventing the erasure of smaller groups.

When the OMB released updated SPD 15 standards in March 2024, CHIS became the first major state population survey to implement them. For this alignment, CHIS combined the race and ethnicity questions, added the new MENA category, and began collecting more detailed ethnic subgroup information within each racial category. Recognizing that federal minimums represent floors, not ceilings, CHIS enhanced the standards with additional categories for locally meaningful subgroups to better represent California's diverse populations. While SPD 15 recommends including the six largest ethnic subgroups within each racial category, CHIS collects more detailed information on smaller communities. For example, CHIS expanded AIAN disaggregation to include Indigenous Mesoamerican identities (Aztec and Maya) , ahead of the passage of California's Latino disaggregation law. By meeting federal minimums, CHIS maintains comparability with national data to allow tracking of disease burden trends; and, by exceeding those minimums, CHIS serves California's specific demographic diversity.

Survey data (numerators) must align with population estimates (denominators) to calculate rates and prevalence and ensure that data are representative of the population. When different systems use incompatible measures and category definitions, data can become unusable for population health surveillance. Creating comparable measures is straightforward when the categories included across sources can be nested hierarchically. For example, although CHIS lists a greater number of Asian ethnic subgroups as response categories within the broader Asian categories, these responses can be combined into the broader Asian category to allow for comparison with the aggregated Asian category reported in ACS population estimates. In this way, the more detailed CHIS data elaborate on rather than conflict with federal standards. But when the boundaries between racial categories are contested rather than nested, harmonization becomes more difficult. The classification of the Armenian population in California under AB 91 described above illustrates the challenge. Under coding guidance proposed by the US Census Bureau for the 2030 Census, Armenian people are classified as White, while California's AB 91 requires Armenian people to be classified as part of the MENA grouping. This creates a situation where state and federal standard definitions diverge.

The CHIS resolves the divergence between federal and state reporting by utilizing a “collect granular, report standard” methodology. This approach balances strict federal compliance with the flexibility required by evolving state standards through two key mechanisms:

- Granular collection: CHIS captures detailed ethnic subgroup write‐ins (e.g., Armenian), preserving authentic self‐identification and high data utility.
- Standardized weighting: To maintain federal consistency, these subgroups are mapped to broader racial categories (e.g., coding Armenian respondents as “White”) during initial data processing and weighting.

This dual‐layer system creates a vital bridging methodology for the implementation of AB 91. Because the underlying subgroup data remain intact, researchers can identify and reassign specific populations across different classification systems regardless of their primary federal race variable.^40^ Ultimately, this preserves the integrity of the data while allowing California to adapt to new legislative mandates without losing historical comparability.

This dual classification enables researchers to produce two types of estimates: one showing trends in health outcomes based on consistently defined populations, and another showing how changes to the racial classification system affect estimates for prevalence rates. This bridging capability is particularly valuable for Latino populations, where combining the race and Hispanic origin questions (rather than asking them separately) changes the distribution of self‐identified race.

The path forward requires ongoing dialogue between critical decision‐makers. CHIS actively engages with the California Department of Finance and California Department of Public Health to build common understanding about population definitions. The California Department of Finance is currently developing work‐arounds to address denominator challenges—producing multiple population estimate series using different classification schemes or providing bridging estimates that allow researchers to choose the denominator that matches their numerator definitions. These innovations require coordination across state agencies and transparency about which estimates are used for which purposes.

**Figure 2:**
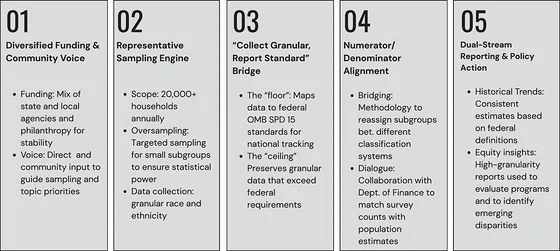
CHIS Playbook on Data Resilience

Figure 2 illustrates the core components of the CHIS data resilience framework.

## Enhancing Data Collection Beyond Race and Ethnicity

While much of the data disaggregation movement has focused on race and ethnicity, building comprehensive state data infrastructure also requires expanding data collection to capture other dimensions of identity that shape health outcomes. Two areas where states have made significant progress are SOGI data and disability data. These efforts offer important lessons for states seeking to build equity‐centered data systems.

Colorado passed HB 22‐1157 in 2022, requiring the Colorado Department of Public Health and Environment to collect and report disability data alongside race, ethnicity, and SOGI data.^41^ The bill impacts the state's various data collection systems, for example, surveys like the Behavioral Risk Factor Surveillance System and Healthy Kids Colorado Survey, and programs including birth/death registries and the Special Supplemental Nutrition Program for Women, Infants, and Children program. SOGI data have long been collected by the Colorado Health Access Survey (CHAS), implemented by the nonprofit Colorado Health Institute, which receives some support from the Colorado Department of Public Health and Environment, since 2009. The CHAS has asked respondents about SOGI, and it collects granular data on race and ethnicity. The survey's large sample size of approximately 10,000 households enables analyses across demographic subgroups and geographic regions.

Oregon similarly has emerged as a leader in the collection of disability data, in addition to its collection of SOGI and detailed race and ethnicity data, through legislative mandates requiring standardized disability data collection across state agencies. Oregon's REALD standards, established through HB 2134 in 2013, require the OHA and Department of Human Services to implement standardized data collection that includes disability status, while companion SOGI standards ensure collection of sexual orientation and gender identity data alongside these other demographics.^27^ During the COVID‐19 pandemic, this framework proved its value when Oregon required all health care providers to collect and report disability status data in all COVID‐19 health encounters, enabling the state to identify and address disparities affecting individuals with disabilities. A key rationale for both states’ legislation was standardizing disability data collection across state departments and organizations, ensuring consistent data to address health inequities, an approach aligned with the Affordable Care Act's emphasis on data collection for underserved populations.

Both SOGI and disability data collection face challenges similar to those documented for race and ethnicity disaggregation: lack of data standardization, the need for training on appropriate data collection methods, ensuring accessibility of data collection processes, and the importance of community engagement in designing data systems.

## Enhancing Data Collection Beyond Traditional Public Health Data: State‐Level Claims Data as Infrastructure

State health care claims data systems, including all‐payer claims databases (APCDs), can be another component of state data infrastructure for understanding disease burden. APCDs aggregate medical, pharmacy, and often dental claims from public and private payers into common, longitudinal data sets, typically including eligibility, diagnosis, procedure, and payment information for most insured residents.^42^ These data allow states to track the incidence and prevalence of treated conditions, patterns of service use, and spending across payers and over time, which is especially valuable for conditions that are undercounted in traditional surveillance data or fragmented across multiple programs.​^43^

When combined with robust demographic fields, APCDs can support equity‐focused analyses of disease burden.^44^ Studies find that race and ethnicity fields in APCDs are often incomplete or inconsistent, particularly for commercially insured populations, leading to large shares of “unknown” and limiting the ability to measure disparities without additional data collection, linkage, or imputation methods.​​^45^ Some states now require payers to submit race and ethnicity variables aligned with federal standards and are beginning to implement collection of more granular ethnic subcategories in response to the recent update to OMB standards. Colorado's APCD, for example, requires payers to submit race and ethnicity data and publishes summary statistics on completeness and patterns of missingness.^46^ Massachusetts redesigned APCD member files to include race, ethnicity, language, disability status, and SOGI starting in 2026.^47^

These experiences illustrate both the potential and limitations of administrative claims data as part of state infrastructure for equity‐centered surveillance.^44^, ^48^ Claims data provide large coverage of insured populations and rich information on diagnoses, procedures, and costs, but equity analyses are constrained when demographic fields are treated as optional or are not standardized and enforced. In addition, APCDs are not designed to capture the entire population and typically do not include those without health insurance or those receiving health care through the Department of Veterans Affairs and Indian Health Service. States seeking to use APCDs to monitor disparities in disease burden will need not only technical investments but also governance mechanisms that mandate high‐quality race, ethnicity, disability, and SOGI data collection and that align APCD standards with survey and vital statistics systems.

## Privacy, Data‐Sharing Standards, and Governance

Despite the benefits of data that are disaggregated and shared across sectors, including enabling robust population‐level analysis that allows deep dives into disparities by various demographic characteristics, there are accompanying risks. These risks include privacy disclosure through improper access, misinterpretation of administrative data collected for nonresearch purposes, replication of structural racism embedded in historical data collection practices, and potential harm to individuals through biased treatment or punitive action.^2^ Building state data infrastructure requires governance frameworks that address the legal, ethical, and technical dimensions of data sharing and privacy.^49^ As the federal data landscape shifts, states must develop their own standards for how data are shared across agencies, how privacy is protected, and how communities are engaged in decisions about data use.

Work by Actionable Intelligence for Social Policy provides guidance that states can adopt to build equity‐centered data governance.^50^ The policies and procedures that determine how data are managed, used, and protected are essential for cross‐sector data efforts. Rather than leaving decisions to individual data custodians, governance should involve multiple stakeholders: those with decision‐making authority, data stewards responsible for metadata and access policies, data custodians responsible for technology, and community members.

Multiple layers of data protection are required; experience from electronic health record infrastructure‐building suggests protections include legal safeguards such as data‐sharing agreements and data use licenses; technical safeguards such as encryption, secure servers, and deidentification protocols; and procedural and physical safeguards such as staff training and locked facilities.^51^ Federal statutes including the Health Insurance Portability and Accountability Act (HIPAA), the Family Educational Rights and Privacy Act, and 42 CFR Part 2 (Confidentiality of Substance Use Disorder Patients Records) establish baseline requirements, but state laws may exceed these federal protections.^52^ For example, California's Confidentiality of Medical Information Act creates stricter rules than HIPAA, requiring patient authorization for disclosures that HIPAA would permit without patient consent; California also classifies patients’ place of birth and immigration status as protected medical information under SB 81.^53^ As states build data infrastructure in the absence of robust federal coordination, attention to privacy, governance, and standards becomes even more critical. Without these foundational elements, the technical capacity to collect disaggregated data will not translate into the analytical capacity to understand health disparities or the trust required to sustain data‐sharing partnerships over time.

Protecting health data requires more than just high‐tech security; it requires strong laws and legal resources such as the legal handbook on data disaggregation from the Network for Public Health Law.^54^ Right now, the federal government is attempting to access state data for new and potentially harmful reasons, notably surveillance or immigration enforcement. To prevent this, states must create data firewalls—legal protections that strictly block the federal government from seeing health records. Without these visible safeguards, people will be too afraid to participate in research. For a major effort like the CHIS, ensuring the state has total control over its data is necessary to get marginalized groups to trust the process and share their information.

## Data Democratization

Data infrastructure serves health equity only if the data it generates are accessible to those who need them. Data democratization ensures communities have meaningful access to data about themselves. As advocates in multiple states have emphasized, during the COVID‐19 pandemic, communities struggled to show health officials how they were disproportionately impacted due to lack of disaggregated data. As a result, invisibility in data translated directly to invisibility in resource allocation. State data infrastructure can embed democratization as a core design principle, as demonstrated by data sources such as CHIS and the California Open Data Portal.^55^ Similarly, the UCLA NHPI Data Policy Lab addresses the historical invisibility of NHPI communities by providing granular, disaggregated data dashboards that have expanded from state‐level mortality tracking for NHPI communities to displaying county‐level social determinants of health.^9^, ^56^, ^57^

Public dashboards offering interactive access to health estimates enable community organizations, journalists, and local health officials to generate customized analyses without statistical software expertise. For example, California Department of Public Health maintains webpages with disaggregated data including death rates by detailed race and ethnicity categories. Oregon's Health Authority has awarded grants to community organizations to facilitate education about the importance of detailed data collection.

Beyond state‐level dashboards, data can and should be disseminated and democratized at even more local levels. One such example is the City Health Dashboard, developed by researchers at New York University Langone Health's Department of Population Health in 2018 with support from the Robert Wood Johnson Foundation, which provides data on health and its drivers for over 1,100 US cities across all 50 states. The dashboard offers city and census tract‐level data on metrics spanning health outcomes, social and economic factors, health behaviors, physical environment, and clinical care, hosted on a free, interactive website with data available for download. The complementary Congressional District Health Dashboard, launched in 2023, extends this model to all 435 congressional districts. This model of localized data is also exemplified by regional initiatives like Community Information Now (CI:Now) in San Antonio, Texas, which provides neighborhood‐level data to support community members with the evidence needed to address local disparities.^58^ These tools are designed for use by community members, local health officials, advocates, and journalists who may lack statistical software expertise but need access to local data. A framework for dissemination tools should include a focus on actionable decision support, away from passive monitoring, which means the tools should be collaborative, timely, culturally relevant, and have a user‐centered approach that ensures data are meaningful.^59^

## Recommendations

Building equity‐centered data infrastructure requires coordinated action across multiple sectors. While we argue state governments must lead with policy and investment, there are important roles for researchers, philanthropic organizations, and community organizations in creating data ecosystems. The following recommendations briefly outline how each sector can contribute to the shared goals we discussed in each section (Table 1).

**Table 1:** Recommendations for Building Equity‐Centered Data Infrastructure by Sector

Playbook Sections	Government	Researchers	Philanthropy	Community
1. Building state data infrastructure	Coordinate denominator data; establish coordination offices and cross‐agency workgroups.	Strengthen partnerships with state health departments; offer technical expertise in systems.	Fund multistate coordination mechanisms to share best practices and technical standards.	Build coalitions with advocacy groups to support state‐level legislation for infrastructure.
2. CHIS model	Support population health surveys with multilanguage/multimode administration and adequate sample sizes.	Provide expertise in survey design, bridging methodologies, and complex sampling.	Treat survey development as a foundational investment; fund enhancements to existing surveys.	Sustain engagement with administrators to ensure data instruments reflect community priorities.
3. Beyond race and ethnicity	Identify and implement critical measures for sexual orientation and gender identity and disability status to track disease burden.	Integrate comprehensive data equity competencies into public health education and training.	Fund data modernization efforts specifically aimed at expanding standards beyond race and ethnicity.	Monitor policy implementation to ensure instruments are updated and used for program modifications.
4. Claims data (all‐payer claims databases)	Invest in a specialized informatics and data science workforce to manage complex systems.	Offer expertise in linkage methodologies to connect claims data with social drivers of health.	Fund infrastructure for data interoperability and the harmonization of disparate data sets.	Develop relationships with agency staff to ensure analysis translates into health care improvements.
5. Privacy and governance	Create governance structures with community representation; adopt interoperability standards.	Convene state learning collaboratives to share practical, privacy‐preserving solutions.	Support the development of state‐level data standards and legal frameworks for data exchange.	Actively participate in governance structures to represent community interests in privacy and access.
6. Data democratization	Develop and maintain public dashboards to ensure data transparency for all stakeholders.	Build sustained relationships with community organizations to ensure findings reflect local priorities.	Fund community data literacy initiatives to empower organizations in advocacy and design.	Use public data tools to monitor outcomes and ensure policies are modified based on findings.

## Conclusion

Addressing disparities in disease burden, mortality, and life expectancy requires data infrastructure that makes population health information visible for and accessible by all communities. The disruption to federal data systems documented throughout 2025, which included systematic alteration of demographic data, discontinuation of critical surveys, and erosion of operational and coordination capacity, threatened this visibility but also creates opportunity for state leadership.

Building resilient, state‐driven health data infrastructure is no longer a matter of administrative preference; it is a necessity for public health survival in a volatile federal landscape. This paper outlines a playbook for states to move beyond federal floors, using technical architecture, shared standards, and data democratization to make every community visible. By treating data equity as a foundational investment rather than an afterthought, states can ensure that the progress made in identifying and addressing disparities is not erased by shifts in federal policy or terminology.

The goal is not 50 incompatible state systems but an ecosystem of equity‐centered data systems that are more responsive, more granular, and more community‐accountable than centralized federal systems alone could achieve. The populations most affected by disparities in disease burden and life expectancy are often those most likely to be invisible in health data. State‐led infrastructure, built through community partnership and held accountable to local populations, offers the possibility of data systems that truly serve health equity and enable policymakers to meet the challenges ahead.

## Funding/Support

This work was conducted by members of the Data Equity Center (DEC) at the UCLA Center for Health Policy Research. The DEC is supported by the Robert Wood Johnson Foundation under a grant entitled “Helping ensure the inclusion of historically underserved communities in data ecosystems.”

## Conflict of Interest Disclosures

None
